# Bleeding outcomes and factor utilization after switching to an extended half-life product for prophylaxis in haemophilia A in Austria

**DOI:** 10.1038/s41598-021-92245-5

**Published:** 2021-06-21

**Authors:** Cihan Ay, Clemens Feistritzer, Joachim Rettl, Gerhard Schuster, Anna Vavrovsky, Leonard Perschy, Ingrid Pabinger

**Affiliations:** 1 Clinical Division of Haematology and Haemostaseology, Department of Medicine I, Medical University of Vienna, Waehringer Guertel 18-20, 1090 Vienna, Austria; 2 Department of Internal Medicine V-Haematology and Oncology, Medical University Innsbruck, Innsbruck, Austria; 3 Department for Internal Medicine and Haematology and Medical Oncology, Klinikum Klagenfurt a.W., Klagenfurt, Austria; 4 Austrian Red Cross, Blood Transfusion Service for Upper Austria, Linz, Austria; 5 Academy for Value in Health, Vienna, Austria

**Keywords:** Diseases, Haematological diseases

## Abstract

To prevent bleeding in severe haemophilia A [SHA, defined as factor VIII (FVIII) activity < 1%] regular prophylactic FVIII replacement therapy is required, and the benefits of factor products with extended half-life (EHL) over traditional standard half-life (SHL) are still being debated. We performed a multi-centre, retrospective cohort study of persons with SHA in Austria aiming to compare clinical outcomes and factor utilization in patients with SHA, who switched from prophylaxis with SHL to an EHL. Data were collected from haemophilia-specific patient diaries and medical records. Twenty male persons with SHA (median age: 32.5 years) were included. The most common reason for switching to the EHL was a high bleeding rate with SHL. Switch to rFVIII-Fc resulted in a significantly decreased annualized bleeding rate (ABR; median difference (IQR): − 0.3 (− 4.5–0); Wilcoxon signed-rank test for matched pairs: Z = − 2.7, p = 0.008) and number of prophylactic infusions per week (− 0.75 (− 1.0–0.0); Z = − 2.7, p = 0.007). Factor utilization was comparable to prior prophylaxis with SHL (0.0 (− 15.8–24.8) IU/kg/week; Z = − 0.4, p = 0.691). In summary, switch to EHL (rFVIII-Fc) was associated with an improved clinical outcome, reflected by ABR reduction, and less frequent infusions, without significantly higher factor usage.

## Introduction

Severe haemophilia A (SHA) is a rare, X-linked genetic bleeding disorder, defined by a baseline coagulation factor VIII (FVIII) activity < 1%. The disease is characterized by the occurrence of spontaneous bleeding events, most commonly affecting joints and muscles^1,2^. Recurrent hemarthrosis leads to joint damage and haemophilic arthropathy, which increases morbidity and decreases quality of life^2^.

To prevent spontaneous bleeding the recommended standard treatment for SHA until recently was regular prophylactic factor replacement therapy via intravenously administered standard half-life FVIII concentrates (SHL) to achieve trough levels > 1%^2,3^. With an average half-life of 8–12 h, regular prophylaxis with SHL usually needs to be administered at least 2–3 times per week^2–5^.

Recently, FVIII products with an extended half-life (EHL), i.e. approximately 1.4–1.6 times greater than SHL, have been approved for use in persons with SHA in Europe, promising lower prophylactic infusion rates and/or higher trough levels^5,6^. Therefore, prophylaxis with EHL holds the potential to improve treatment adherence and/or decrease the annualized bleeding rate (ABR), subsequently raising quality of life, especially for persons with SHA on prophylaxis with difficult venous access or experiencing frequent breakthrough bleeding events^5–9^.

The safety and efficacy of the available EHL has been confirmed in clinical trials^10–13^. Real-world data from studies exploring the effects of persons with SHA switching to EHL show benefits regarding infusion frequency and bleeding outcomes, albeit mostly at higher costs^6,14–19^. However, EHL treatment may still be cost-effective in the long term by reducing bleeding events and improving joint health^20^. Regardless, there are currently no guidelines as to which patients should undergo the switch from SHL to EHL, and despite the fact that potentially all patients could benefit from it the question of who would profit most from the switch is still being debated^4,5^.

In Austria, no data of EHL use in clinical practice are currently available. Therefore, we aimed to investigate the clinical outcomes, drug utilization and costs in patients with SHA who switched from SHL products to efmoroctocog alfa (Elocta), a recombinant FVIII-Fc-fusion-protein (rFVIII-Fc) and the first approved EHL used in clinical practice^6,10^. Such data may help to understand the benefits of EHL outside of clinical trials and optimize their use in everyday clinical practice.

## Materials and methods

### Study design, setting and population

We performed a retrospective, multi-centre cohort study of persons with SHA, who were switched from regular prophylaxis with SHL to prophylaxis with EHL, to investigate clinical outcomes [annualized bleeding rate (ABR) and infusion frequency], drug utilization and costs in a real-world setting in Austria. This analysis was limited to efmoroctocog alfa (Elocta, rFVIII-Fc) as it was the first approved EHL in Austria and thus the only EHL to which a significant number of patients were switched in real-world clinical practice. Data were collected from medical records and, if available, haemophilia-specific diaries from seven different Austrian haemophilia care centres and treatment sites (Clinical Division of Haematology and Haemostaseology, Department of Medicine I, Medical University of Vienna, Vienna, Austria; Department for Internal Medicine and Haematology and Medical Oncology, Klinikum Klagenfurt a.W., Klagenfurt, Austria; Austrian Red Cross, Blood Transfusion Service for Upper Austria, Linz, Austria; Department of Child and Adolescent Health, Department of Paediatrics, Universitätsklinikum St. Pölten, Karl Landsteiner University of Health Sciences Austria, St. Pölten, Austria; Department for Child and Adolescent Health, LKH Bregenz, Bregenz, Austria; Department of Internal Medicine V—Haematology and Oncology, Medical University Innsbruck, Innsbruck, Austria; Department of Child and Adolescent Health, Medical University Innsbruck, Innsbruck, Austria).

All persons with SHA on prophylaxis who were switched until December 2018 and who had been treated with the EHL for at least 3 months and complete data documentation were eligible for this analysis. Patients on on-demand treatment directly prior to receiving prophylaxis with the EHL were excluded. From the available data sources, we extracted the following information: patients’ characteristics, reasons for switch to EHL, inhibitor development, prophylactic regimens, factor use and bleeding events (to calculate the ABR) during prophylaxis with the SHL and after switch to prophylaxis with the EHL. Furthermore, we calculated and compared factor usage in IU/kg/week and drug costs per month of SHL and EHL prophylaxis. To perform this comparison, data were collected for the 6 months preceding the switch and for at least 3 months under the EHL regimen. We calculated the drug costs per month for the respective SHL factor product used as well as the EHL (rFVIII-Fc), according to costs indicated in an online index of medicinal products in Austria (“Warenverzeichnis online des Österreichischen Apotheker-Verlags”, see supplementary table)^21^.

### Statistical analysis

We performed descriptive statistics using absolute frequencies, percentages, arithmetic mean, standard deviation, range, median and interquartile range (i.e. range between 25th-percentile and 75th percentile), where appropriate.

Differences in injections per week, factor consumption, costs and ABR were evaluated for all patients with available datapoints both pre and post switch, using Wilcoxon signed-rank tests for matched pairs. We used Spearman’s rank correlation coefficient to calculate correlations between parameters. A p-value less than 0.05 was considered statistically significant.

Patients with incomplete data sets where excluded from analyses requiring the respective missing variable, however, were still included in analyses for which they had applicable data.

Statistical analyses were performed using IBM SPSS Statistics (IBM, New York, USA).

### Ethics declarations

The study was conducted in accordance with GCP guidelines and the Declaration of Helsinki and approved by the Ethics Committee of our institution (Ethics Committee of the Medical University of Vienna, EK Nr: 2093/2018). As this study was retrospective in nature, informed consent was not obtained. This decision was approved by the Ethics Committee of the Medical University of Vienna.

## Results

### Description of study cohort and pre switch data

Twenty-three male persons with SHA at participating study sites had been switched to EHL [i.e. rFVIII-Fc, efmoroctocog alfa (Elocta)], and were then on a regular prophylaxis for at least 3 months. One subject had to be excluded, as no data during prior prophylaxis with the SHL was available. Two subjects were excluded as they had performed on-demand treatment with an SHL before switching to EHL. Therefore, the final study cohort consisted of 20 subjects (median age 32.5 years, IQR: 25.3–42.6, range: 9–75).

We collected data on treatment patterns and bleeding outcomes for the 6-month period preceding the switch to EHL. The most common SHL factor products used pre switch were rurioctocog alfa (Advate, 8/20 or 40.0%), followed by recombinant octocog alfa (Helixate) (5/20 or 25.0%). The most common prophylactic infusion frequency pre switch was every other day (7/20 or 35.0%), followed by 3×/week and 2×/week (each 5/20 or 25%); the median (IQR) number of prophylactic infusions per week was 3.0 (2.0–3.5); the calculated median (IQR) prophylactic factor usage was 73.7 (58.8–95.9) IU/kg/week (n = 19); The median (IQR) ABR during prophylaxis with the SHL in the 6 months prior to switch was 1.0 (0.0–6.0), the median (range) number of surgeries (including dental interventions) was 0.0 (0–2).

A description of the study cohort and pre switch data can be found in Table 1.

**Table 1:** Description of the study cohort and pre switch (i.e. 6-month period of SHL prophylaxis before switch to EHL) data (n = 20).

**Demographics**	
Median (IQR) age, years	32.5 (25.3–42.6)
< 18 years, n (%)	2 (10.0)
Mean ± SD weight (kg)	73.0 ± 18.3
Mean ± SD height (cm)^a^	171.0 ± 13.9
Mean ± SD BMI^a^	24.4 ± 4.2
Median (range) time EHL was used (days)^b^	392 (102–938)
**Factor product pre switch, n (%)**	
Rurioctocog alfa (Advate)	8 (40.0)
Octocog alfa, recombinant (Helixate)	5 (25.0)
Octocog alfa, recombinant (Kogenate)	3 (15.0)
Octocog alfa, plasma-derived (Beriate)	2 (10.0)
Octocog alfa, plasma-derived (Haemate)	1 (5.0)
Moroctocog alfa (Refacto)	1 (5.0)
**Prophylactic infusion frequency with SHL pre switch, n (%)**	
1×/week	1 (5.0)
1–2×/week	1 (5.0)
2×/week	5 (25.0)
2–3×/week	0 (0.0)
3×/week	5 (25.0)
Every other day	7 (35.0)
Unknown/variable	1 (5.0)
**Bleeding outcomes during prophylaxis with SHL**^**a**^	
Median (IQR) ABR	1.0 (0.0–6.0)
Mean (± SD) ABR	6.4 (12.2)
Median (range) number of surgeries	0 (0–2)

Description of the study cohort including demographics, factor products and infusion frequency pre switch (during prophylaxis with SHL).

^a^Here, n = 19, as values were not recorded, and therefore not available for one subject.

^b^Here, n = 18, as values were not recorded, and therefore not available for two subjects.

### Post switch data

Reasons for subjects switching to the EHL were analysed and categorized. The most common primary reason was a high number of bleeding events (5/20 or 25%). The primary and secondary reasons for the switch to EHL treatment are summarized in Table 2.

**Table 2:** Reasons for switching to EHL (n = 20).

Primary reasons	n (%)
**Better efficacy**	**7 (35.0)**
Improved efficacy expected	2 (10.0)
High number of bleeds with SHL	5 (25.0)
**Prolonged half-life**	**4 (20.0)**
Extended half-life	2 (10.0)
Reduced prophylactic infusion frequency	2 (10.0)
**Convenience/patient preference**	**4 (20.0)**
Patient preference	2 (10.0)
Difficult venous access	2 (10.0)
**Other**	**5 (25.0)**
Previous SHL no longer available	2 (10.0)
Study participation	2 (10.0)
Inhibitors	1 (5.0)
**Secondary reasons**	**n (%)**
No secondary reason given	12 (60.0)
Extended half-life	2 (10.0)
Reduced prophylactic infusion frequency	2 (10.0)
Efficacy	1 (5.0)
Pain	1 (5.0)
Difficult venous access	1 (5.0)
Depression	1 (5.0)

Summary of the primary and secondary reasons for persons with SHA switching from SHL to EHL (rFVIII-Fc, Elocta).

We collected data for at least 3 months under EHL treatment. All subjects were using efmoroctocog alfa (Elocta). The most common prophylactic infusion frequency post switch was 2×/week (8/20 or 40.0%); the median (IQR) number of prophylactic infusions per week was 2.0 (2.0–2.3); the calculated median (IQR) prophylactic factor usage was 73.6 (58.0–85.4) IU/kg/week; The median (IQR) ABR during EHL prophylaxis was 0.0 (0.0–1.5), the median (range) number of surgeries (including dental interventions) was 0 (0–1). We found no statistically significant correlation between prescribed weekly factor dosage and ABR post switch (Spearman correlation coefficient: r_s_ = − 0.404, p = 0.086). None of the patients developed inhibitors after switching to efmoroctocog alfa (Elocta). Table 3 provides a summary of the post switch data.

**Table 3:** Post switch (EHL) data (n = 20).

**Factor product post switch, n (%)**	
Efmoroctocog alfa (Elocta)	20 (100.0)
**Prophylactic infusion frequency post switch, n (%)**^**a**^	
1×/week	1 (5.0)
1–2×/week	2 (10.0)
2×/week	8 (40.0)
2–3×/week	6 (30.0)
3×/week	0 (0.0)
Every other day	2 (10.0)
Unknown/variable	1 (5.0)
**Bleeding outcomes under EHL**^**a**^	
Median (IQR) ABR	0.0 (0.0–1.5)
Mean (± SD) ABR	2.3 (6.2)
Median (range) number of surgeries	0 (0–1)

Factor products, prophylactic infusion frequency and bleeding outcomes for persons with SHA after switching to EHL (Elocta, rFVIII-Fc).

^a^Here, n = 19, as values were not recorded, and therefore not available for one subject.

### Comparisons of SHL vs. EHL with regard to bleeding outcomes, infusion frequency, factor usage and costs

To compare bleeding outcomes, we calculated the difference in ABR pre and post switch. Ten (50.0%) subjects had a lower ABR, for eight (40.0%) subjects, there was no difference in ABR and only one (5.0%) subject had a higher ABR after the switch to EHL (rFVIII-Fc). For one subject (5.0%), these calculations could not be carried out because of missing data. The median (IQR) difference in ABR per patient after switch from SHL to EHL was − 0.3 (− 4.5–0), and the Wilcoxon signed-rank test for matched pairs showed a statistically significant decrease in ABR after the switch from SHL to EHL treatment (n = 19; Z = − 2.7; p = 0.008).

Next, we compared prophylactic infusion frequency and found that twelve (60.0%) subjects had a lower infusion frequency per week, four (20.0%) had no change and two (10.0%) subjects had a higher infusion frequency after the switch to EHL (rFVIII-Fc). Two (10.0%) subjects had an incomplete data set with respect to details on prophylactic infusions. The median (IQR) difference in the number of prophylactic infusions per week was − 0.75 (− 1.0–0.0) after switching to EHL (Wilcoxon signed-rank test for matched pairs: Z = − 2.7; p = 0.007).

Furthermore, we compared prophylactic factor usage with SHL and EHL. Eight (40.0%) subjects had a decreased and seven (35.0%) subjects had an increased factor usage after the switch to EHL. In three (15.0%) subjects, there was no difference in factor usage. Two subjects (10.0%) had incomplete datasets in this regard. The median (IQR) difference in prophylactic factor usage was 0.0 (− 15.8–24.8) IU/kg/week (Wilcoxon signed-rank test for matched pairs: Z = − 0.4; p = 0.691).

Lastly, we compared costs of factor usage pre and post switch. To this end, we calculated the costs per month for the respective SHL and the EHL (rFVIII-Fc) based on costs indicated in an online index of medicinal products in Austria (“Warenverzeichnis online des Österreichischen Apotheker-Verlags”, see supplementary table)^21^. We found that for seven (35.0%) subjects, costs per month had decreased and for eleven (55.0%) subjects, costs had increased. Two (10.0%) subjects had incomplete data sets. The median (IQR) difference in costs per month was € + 600 (− 1800–4050) after the switch (Wilcoxon signed-rank test for matched pairs: Z = − 0.675; p = 0.500).

**Figure 1:**
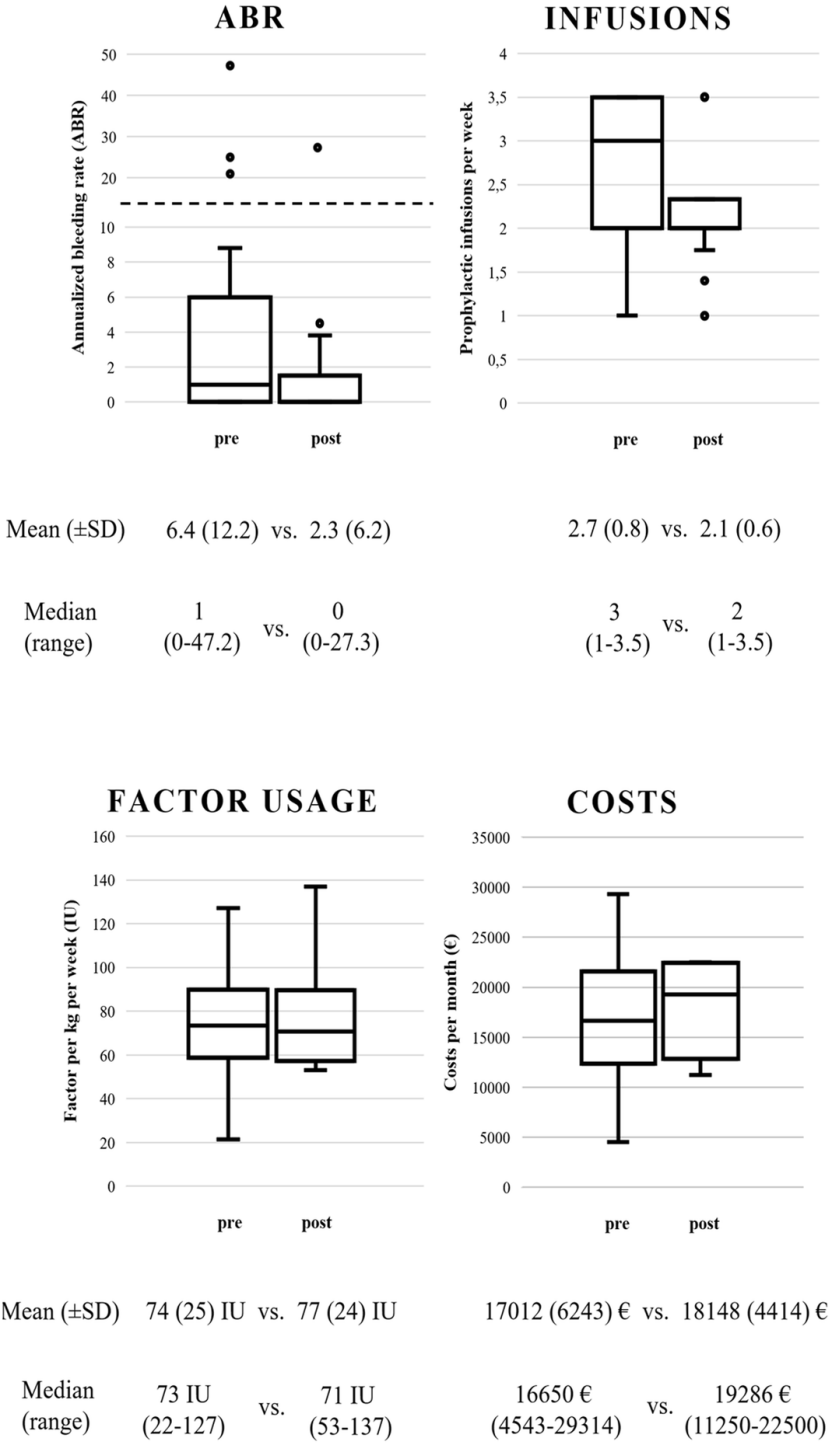
SHL and EHL (rFVIII-Fc) comparisons (n = 19/18/18/18). Comparisons of bleeding outcomes (ABR, n = 19), infusion frequency (n = 18), factor usage (n = 18) and costs per week pre and post switch (n = 18) from SHL to EHL (rFVIII-Fc) in persons with SHA. The Y-axis in the ABR graph was split for better representability (indicated by the dashed line). It is important to note that only subjects with complete data sets for the respective analyses were included.

Figure 1 shows a summary of the SHL and EHL (rFVIII-Fc) comparisons using box plots.

## Discussion

In a retrospective, multi-centre cohort study of persons with SHA switching from prophylaxis with SHL to an EHL (rFVIII-Fc, Elocta), we analysed and compared treatment patterns in terms of bleeding outcomes, infusion frequency and factor usage, and costs before and after the switch. While real-world data regarding EHL is becoming increasingly available for other countries, this is the first analysis of its kind in Austria^6,14–20,22^.

We found that the most common reason for switching from SHL to EHL (rFVIII-Fc) was the expected improved efficacy. Reduced prophylactic infusion frequency and convenience for patients were other reasons for switch to EHL in our study cohort. This observation is in line with the expectations of EHL of persons with haemophilia (PWH) in other studies, although reduced infusion frequency seemed to be of greater interest to PWH than efficacy^22,23^. It has also been reported that the discussion about switching to an EHL was more likely to be brought up by a physician, not the PWH, as information regarding EHL was largely unknown to patients^22,23^. In our study, we could not entirely distinguish whether the reasons for switching to EHL were the treating physicians’ or the patients’ preference. Our findings further stress that PWH should be continuously educated about haemophilia treatment to best address patients’ needs^23–25^.

The switch to the EHL has met the expectations based on the primary reasons for switching, as we observed a statistically significant reduction in ABR and prophylactic infusion frequency per week after switching from SHL to EHL. This was also found in other publications from high-income countries investigating rFVIII-Fc, reporting higher trough levels, lower infusion frequencies and improved bleeding outcomes^6,17–19,26^.

However, we found no statistically significant change in factor usage and costs, in contrast to other real-world data, indicating that a decrease in factor consumption is possible^14,17–19,26^. The difference in our findings could be explained by the low-dose prophylactic regimen for SHL in Austria^27^.

Still, currently available publications have reported increased costs of treatment with EHL compared to SHL^14,16^. This issue is still being debated as the benefits in ABR reduction, and thus improved joint health may positively impact factor usage and cost-effectiveness in the long term^5^. However, it can be challenging to compare changes in costs across studies conducted in different countries as factor product pricing may vary.

Haemophilia treatment is a rapidly developing field; however, all the new therapeutic options are still relatively high-priced^14,16,28–31^. Therefore, the pros and cons should be carefully weighed when considering the transition to a new treatment option. New therapeutic approaches include emicizumab, a bispecific humanized antibody mimicking factor VIII with a half-life of 30 days. It can be applied subcutaneously and may also be used in the presence of inhibitors^32^. Furthermore, gene therapy is another promising treatment approach by providing the affected individual with the respective factor gene via a vector. Thus, it holds the potential of curing haemophilia. However, there is currently no application outside of clinical trials^33^. The data our study provides—showing a reduction in the bleeding rate and infusion frequency without significant increase of costs—should prove useful in the implementation of EHL for prophylaxis in SHA and may help in the decision making in the context of other new treatment options and their respective costs.

Our study has several limitations. Owing to our small sample size, it was difficult to draw strong conclusions. Further studies with a larger sample size and including other EHL products in addition to rFVIII-Fc are required to confirm our results and the benefits of other EHL in Austria. We could not provide pharmacokinetic data (individual half-life and trough levels), given the retrospective design of the study. Also, we had to rely on patient’s diligence to accurately report bleeding events, and based on the available data sources, we were unable to separate different categories and types of bleeds. Furthermore, we had to calculate factor usage and costs for our subjects according to the prophylactic treatment regimen. The actual amount of used factor product may deviate somewhat from our calculations. Thus, we could not take into consideration treatment adherence which was demonstrated to be poorer in adult patients^34^. In addition, we only calculated prophylactic factor usage and costs of infusions for treating bleeding episodes were not taken into consideration. However, bearing in mind the lower ABR under EHL compared to SHL, the overall factor consumption, and consequently, overall costs, may still be lower under EHL than SHL prophylaxis as there were fewer bleeding events and therefore less additional factor usage for treating bleeding episodes under EHL prophylaxis.

In summary, the switch to EHL (rFVIII-Fc) was associated with an improved clinical outcome, reflected by a statistically significant reduction of the ABR, and a lower prophylactic infusion frequency per week, without significantly higher factor usage and costs. Thus, we believe that especially persons with SHA with a high number of bleeds and therefore, we speculate, also those with poor treatment adherence due to difficult venous access may benefit from prophylaxis with EHL. Further research is required to demonstrate the effect of switching persons from SHL to EHL regarding factor usage and total costs. Moreover, additional data on EHL in everyday clinical practice should help establishing their role in the rapidly evolving therapeutic landscape of haemophilia.

## Supplementary Information

Supplementary Information.

## Data Availability

The data set generated for and/or analysed in this study are not publicly available due to medical confidentiality but are available from the first author on reasonable request.

## References

[1] Peyvandi F, Garagiola I, Young G (2016). The past and future of haemophilia: Diagnosis, treatments, and its complications. Lancet.

[2] Srivastava A (2013). Guidelines for the management of hemophilia. Haemophilia.

[3] Pabinger I (2015). Hämophiliebehandlung in Österreich. Wien. Klin. Wochenschr..

[4] Ar MC, Balkan C, Kavaklı K (2019). Extended half-life (EHL) coagulation factors: A new era in the management of haemophilia patients. Turk. J. Hematol..

[5] Chowdary P (2019). Extended half-life recombinant products in haemophilia clinical practice—Expectations, opportunities and challenges. Thromb. Res..

[6] Peyvandi F (2019). Real-life experience in switching to new extended half-life products at European haemophilia centres. Haemophilia.

[7] Graf L (2018). Extended half-life factor VIII and factor IX preparations. Transfus. Med. Hemother..

[8] Kumar R, Dunn A, Carcao M (2016). Changing paradigm of hemophilia management: Extended half-life factor concentrates and gene therapy. Semin. Thromb. Hemost..

[9] Lambert T (2018). Practical aspects of extended half-life products for the treatment of haemophilia. Ther. Adv. Hematol..

[10] Mahlangu J (2014). Phase 3 study of recombinant factor VIII Fc fusion protein in severe hemophilia A. Blood.

[11] Hampton K (2017). First report on the safety and efficacy of an extended half-life glycoPEGylated recombinant FVIII for major surgery in severe haemophilia A. Haemophilia.

[12] Reding MT (2017). Safety and efficacy of BAY 94–9027, a prolonged-half-life factor VIII. J. Thromb. Haemost..

[13] Curry N (2019). Once-weekly prophylaxis with glycoPEGylated recombinant factor VIII (N8-GP) in severe haemophilia A: Safety and efficacy results from pathfinder 2 (randomized phase III trial). Haemophilia.

[14] Chhabra A (2018). Real-world analysis of dispensed international units of coagulation factor VIII and resultant expenditures for hemophilia A patients: A comparison between standard half-life and extended half-life products. Manag. Care.

[15] Simpson ML, Desai V, Maro GS, Yan S (2020). Comparing factor use and bleed rates in U.S. Hemophilia A patients receiving prophylaxis with 3 different long-acting recombinant factor VIII products. J. Manag. Care Spec. Pharm..

[16] Kim HK, Peral C, Rubio-Rodríguez D, Rubio-Terrés C (2020). Cost of patients with hemophilia A treated with standard half-life or extended half-life FVIII in Spain. Expert Rev. Pharmacoecon. Outcomes Res..

[17] Wang C, Young G (2018). Clinical use of recombinant factor VIII Fc and recombinant factor IX Fc in patients with haemophilia A and B. Haemophilia.

[18] Tagliaferri A (2019). Optimising prophylaxis outcomes and costs in haemophilia patients switching to recombinant FVIII-Fc: A single-centre real-world experience. Blood Transfus..

[19] Keepanasseril A (2017). Switching to extended half-life products in Canada—Preliminary data. Haemophilia.

[20] Bullement A, McMordie ST, Hatswell AJ, Li N, Wilson K (2020). Cost-effectiveness analysis of recombinant factor VIII Fc-fusion protein (rFVIIIFc) for the treatment of severe hemophilia A in Italy incorporating real-world dosing and joint health data. PharmacoEcon. Open.

[21] Österreichische Apotheker-Verlagsgesellschaft m.b.H. Warenverzeichnis online der Österreichischen Apothekerkammer. *Warenverzeichnis Apoverlag* warenverzeichnis.apoverlag.at (2019).

[22] Khair K (2019). HOw Patients view Extended half-life products: Impressions from real-world experience (The HOPE study). Haemophilia.

[23] von Mackensen S (2017). Haemophilia patients’ unmet needs and their expectations of the new extended half-life factor concentrates. Haemophilia.

[24] Lindvall K, Colstrup L, Loogna K, Wollter IM, Grönhaug S (2010). Knowledge of disease and adherence in adult patients with haemophilia. Haemophilia.

[25] Lane S (2013). What should men living with severe haemophilia need to know? The perspectives of Canadian haemophilia health care providers. Haemophilia.

[26] Brennan Y, Parikh S, McRae S, Tran H (2020). The Australian experience with switching to extended half-life factor VIII and IX concentrates: On behalf of the Australian Haemophilia Centre Directors’ Organisation. Haemophilia.

[27] Ay C, Perschy L, Rejtö J, Kaider A, Pabinger I (2020). Treatment patterns and bleeding outcomes in persons with severe hemophilia A and B in a real-world setting. Ann. Hematol..

[28] Rodriguez-Merchan EC (2020). The cost of hemophilia treatment: The importance of minimizing it without detriment to its quality. Expert Rev. Hematol..

[29] Chen S-L (2016). Economic costs of hemophilia and the impact of prophylactic treatment on patient management. Am. J. Manag. Care.

[30] Patel AM, Corman SL, Chaplin S, Raimundo K, Sidonio RF (2019). Economic impact model of delayed inhibitor development in patients with hemophilia a receiving emicizumab for the prevention of bleeding events. J. Med. Econ..

[31] Cortesi PA (2020). Cost-effectiveness and budget impact of emicizumab prophylaxis in haemophilia A patients with inhibitors. Thromb. Haemost..

[32] Franchini M (2019). Emicizumab for the treatment of haemophilia A: A narrative review. Blood Transfus..

[33] Perrin GQ, Herzog RW, Markusic DM (2019). Update on clinical gene therapy for hemophilia. Blood.

[34] Mason JA, Parikh S, Tran H, Rowell J, McRae S (2018). Australian multicentre study of current real-world prophylaxis practice in severe and moderate haemophilia A and B. Haemophilia.

