# Supplementary material for: Bleeding outcomes and factor utilization after switching to an extended half-life product for prophylaxis in haemophilia A in Austria
Source: Sci Rep. 2021 Jun 21;11:12967. doi: 10.1038/s41598-021-92245-5 (PMC8217178; doi:10.1038/s41598-021-92245-5)
Supplement: Supplementary file 1 — Supplementary Information. [file 41598_2021_92245_MOESM1_ESM.pdf]

Bleeding outcomes and factor utilization after switching to an extended half-life product for prophylaxis in haemophilia A in Austria

Cihan Ay[1*], Clemens Feistritzer[2], Joachim Rettl[3], Gerhard Schuster[4], Anna Vavrovsky[5], Leonard Perschy[1], Ingrid Pabinger[1]

[*] cihan.ay@meduniwien.ac.at

[1] Clinical Division of Haematology and Haemostaseology, Department of Medicine I, Medical University of Vienna, Vienna, Austria

[2] Department of Internal Medicine V - Haematology and Oncology, Medical University Innsbruck, Innsbruck, Austria

[3] Department for Internal Medicine and Haematology and Medical Oncology, Klinikum Klagenfurt a.W., Klagenfurt, Austria

[4] Austrian Red Cross, Blood Transfusion Service for Upper Austria, Linz, Austria

[5] Academy for Value in Health, Vienna, Austria

Supplementary table: Details and costs of FVIII products

Trade name	Costs per infusion of 1000IU (€)	Costs/IU (€)
Standard half-life factor products (SHL)		
Advate	760,00	0,76
Kogenate	717,00	0,72
Helixate	717,00	0,72
Refacto	716,50	0,72
Haemate	621,30	0,62
Beriate	533,12	0,53
Extended half-life factor products (EHL)		
Elocta	750,00	0,75

Supplementary table containing details of mentioned FVIII products and their costs. This was the basis of calculation for our cost comparison.
Reference: Österreichische Apotheker-Verlagsgesellschaft m.b.H. Warenverzeichnis online der Österreichischen Apothekerkammer. Warenverzeichnis Apoverlag. Published July 2019.
warenverzeichnis.apoverlag.at
